# The contribution of a person-centred model of Lean Six Sigma to the development of a healthful culture of health systems improvement

**DOI:** 10.3389/frhs.2025.1621233

**Published:** 2025-08-18

**Authors:** Seán Paul Teeling, Deborah Baldie, Ailish Daly, Anne Marie Keown, Aileen Igoe, Ciara Dowling, Martin McNamara

**Affiliations:** ^1^ UCD Centre for Interdisciplinary Research, Education & Innovation in Health Systems, School of Nursing, Midwifery & Health Systems, University College Dublin, Dublin, Ireland; ^2^ Mater Transformation, Mater Misericordiae University Hospital, Dublin, Ireland; ^3^ Centre for Person-Centred Practice Research Division of Nursing, School of Health Sciences, Queen Margaret University Drive, Queen Margaret University, Musselburgh, United Kingdom; ^4^ Research and Practice Development, NHS Grampian, Aberdeen, Scotland; ^5^ Patient Safety, Quality & Innovation, Beacon Hospital, Dublin, Ireland; ^6^ Nursing Department, Incorporated Orthopaedic Hospital of Ireland, Dublin, Ireland

**Keywords:** Lean Six Sigma, person-centred care, healthful cultures, healthcare improvement, systems thinking, reflective practice, staff empowerment, sustainable change

## Abstract

**Background:**

A failure to distinguish between person-centredness, person-centred care, and person-centred cultures can result in improvement initiatives focusing solely on improvement initiative metrics and outcomes, excluding the authentic experiences of patients and staff. Building on the foundational work of Dewing and McCormack, we have designed, piloted, and implemented the Person-centred Lean Six Sigma (PCLSS) model in public and private acute and community healthcare settings across Ireland. This model uses Lean Six Sigma, a widely adopted improvement methodology, through a person-centred lens with which improvement practitioners and healthcare staff can inspect their Lean Six Sigma practice and critically evaluate whether, to what extent, and how it is synergistic with person-centred approaches.

**Aim:**

This paper explores the deployment of the PCLSS model across four clinical study sites and examines its alignment with McCance and McCormack's conceptual work on healthful cultures, evaluating its contribution to creating cultures that support sustainable improvement, compassion, and respect.

**Methods:**

The PCLSS model was embedded within a university-accredited education programme for healthcare staff. The model was applied across four distinct healthcare settings in Ireland: a public acute teaching hospital, a private full-service acute hospital, an integrated ophthalmology service bridging hospital and community care, and a public rehabilitation hospital. A case study methodology was used to examine implementation and impact.

**Results:**

Across all four sites, the PCLSS model facilitated improvements in operational efficiency, staff and patient engagement, interprofessional collaboration, and reflective practice. The model supported leadership at all levels, fostered sustainable change, and successfully mapped onto key domains associated with healthful cultures, as articulated in the work of McCance and McCormack.

**Conclusion:**

The PCLSS model represents a sustainable, values-based approach to improvement that aligns operational excellence with person-centred principles. Its application contributes meaningfully to the development of healthful cultures in healthcare organisations.

## 1 Introduction

### 1.1 Background: person-centred care and Lean Six Sigma

Person-centredness continues to be a cornerstone of contemporary healthcare, influencing how care is delivered and experienced and how improvements are made within healthcare systems (1). At its core, it promotes meaningful relationships between all those involved in care. It is defined as “an approach to practice established through the formation and fostering of healthful relationships between all care providers, service users and others significant to them in their lives” (1). Person-centred care is the operational enactment of these values in day-to-day clinical encounters and focuses on planning and delivering care in a way that is meaningful to each individual (2). Person-centred cultures represent the broader organisational embodiment of these principles, reflected in leadership, governance, infrastructure, and values systems (1). Distinguishing between person-centredness, person-centred care, and person-cultures is essential for embedding authentic, sustainable change (3).

Lean is a process improvement methodology that focuses on the elimination of non-value-added (NVA) activities—those steps in a process that do not add value from the perspective of the person receiving or delivering care (3). Examples of the impact of NVA include prolonged wait times for diagnosis, intervention or treatment (4, 5), variable access to sufficient treatment such as physiotherapy or occupational therapy (6), or lack of system oversight of care between acute hospitals and community settings (7). Six Sigma is a data-driven methodology aimed at minimising unwanted process variation that can lead to errors, inconsistencies, or inefficiencies, such as variability in medication administration (8) or delays in moving older persons from acute care to home settings (9).

When combined, Lean Six Sigma (LSS) integrates Lean's reduction of non-value-added activities focus with Six Sigma's variation-minimising rigour to optimise healthcare processes, improve reliability, and enhance patient outcomes (3, 4). It has become one of the most widely adopted methodologies in international healthcare improvement practice (3, 10).

While LSS offers structured methods for problem-solving and process improvement, its application in healthcare has often been technical in focus, sometimes failing to account for the relational and cultural aspects of care (3, 11). When deployed without attention to human values, LSS risks becoming a decontextualised and at times, reductionist-focused toolkit that overlooks the experiences of patients and staff (12). However, reports of recent work at both local and systems levels in healthcare settings (13) demonstrate the potential synergy between LSS and person-centredness when framed intentionally through models such as the Person-centred Lean Six Sigma (PCLSS) model, creating opportunities to embed relational and cultural values at the heart of improvement work (14).

### 1.2 The development of the PCLSS model

The PCLSS model's conceptual foundation is rooted in the idea of “human flourishing”, which can be described as a state in which individuals experience sustained well-being and function at their best (15). This includes resilience—the capacity to adapt and grow following periods of challenge, drawing on positive psychology principles from authors such as Seligman (16).

**Figure 1:**
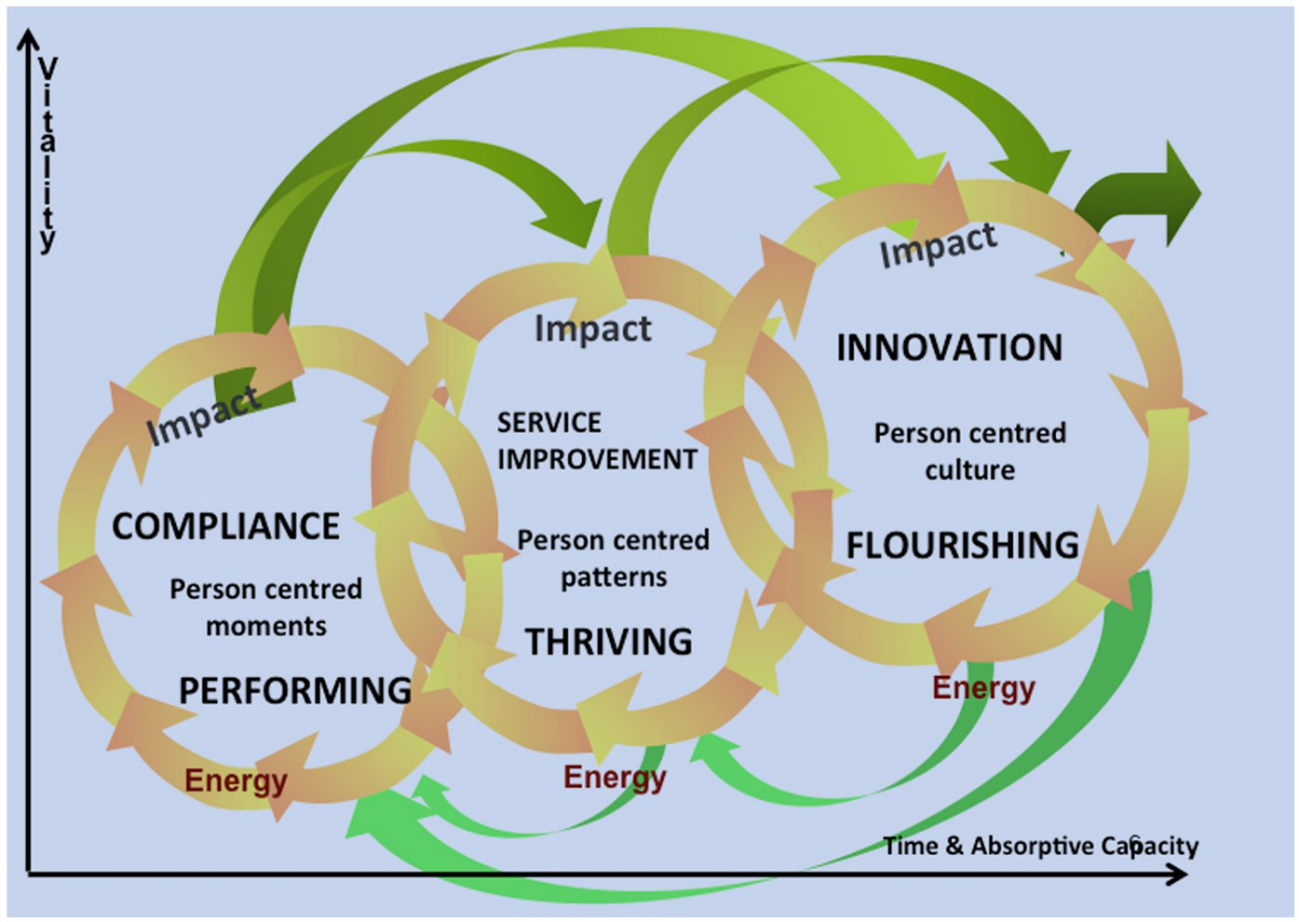
The compliance, service improvement and innovation model (CoSII). This Venn-style model presents three overlapping domains: Compliance, Service Improvement, and Innovation, each aligned with stages of person-centred moments (performing), patterns (thriving), and cultures (flourishing). It originated from a practice-development programme focused on energy and movement as drivers of change. Organisational activities populate different domains: compliance emphasises technical adherence; improvement embodies process-driven engagement; innovation enables flourishing cultures. Axes illustrate how emotional and motivational energies are generated across domains, reinforcing that sustainable person-centred culture requires movement through and across these interlinked spaces. Reproduced with permission from “The Compliance, Service Improvement and Innovation Model (CoSII)” by Brendan McCormack and Tanya McCance.

To support workplaces in enabling human flourishing Dewing et al. (17) and Dewing and McCormack (18) developed the Compliance, Service Improvement and Innovation (CoSII) model (Figure 1). The CoSII model positions service improvement, including Lean Six Sigma, alongside compliance and innovation within a broader person-centred cultural framework. It acknowledges that organisations may fluctuate between these orientations over time, and that culture change is a dynamic, evolving process.

This conceptual foundation laid the groundwork for the development of the Person-centred Lean Six Sigma (PCLSS) model. The CoSII model illustrated that even structured methodologies like Lean Six Sigma can align with person-centred principles when applied intentionally. Building on this insight, Teeling et al. (3, 11) explored the points of synergy and divergence between Lean Six Sigma and person-centred approaches. Through a realist review of the literature and a realist evaluation of real-world practice, they examined how Lean Six Sigma could contribute to the development of person-centred cultures—insights that informed the creation of the PCLSS model (14).

**Figure 2:**
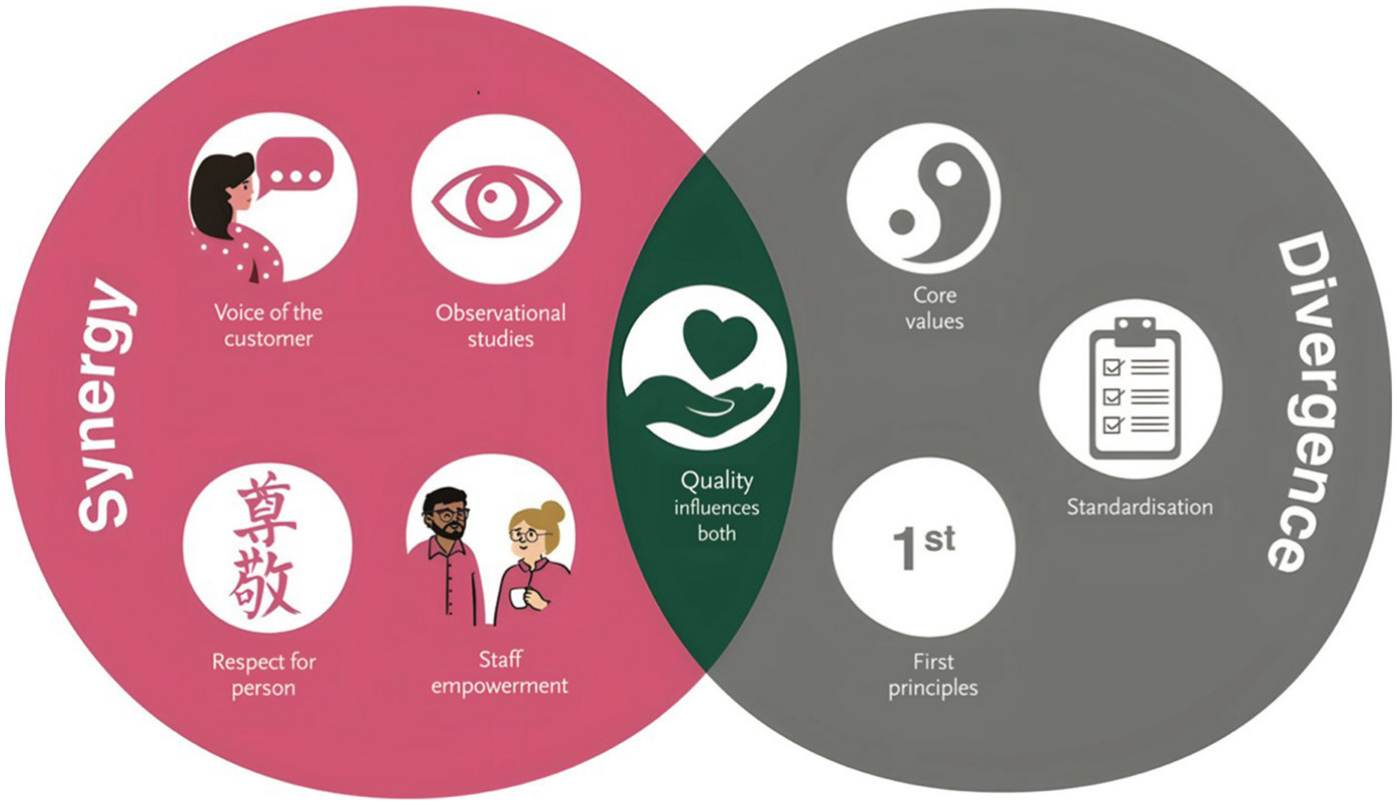
The person-centred Lean Six Sigma model. This circular model presents eight Lean Six Sigma components viewed through a person-centred lens, grouped into three domains: Synergy, Divergence, and Mutual Influence. Voice of the Customer, Respect for Person, Observational Studies (Gemba), and Staff Empowerment are located in Synergy, reflecting strong alignment with person-centred principles. Core Values, Standardisation, and First Principles are positioned in Divergence, where potential tensions may arise. The central overlapping space, containing *Quality as Influencer*, represents the intentional integration of relational and technical logics, drawing on the contrasting perspectives represented in the Synergy and Divergence domains. Acknowledging that understandings of quality are shaped by context, culture, and system priorities, the model avoids collapsing these domains into one. Instead, it foregrounds how quality may serve as a bridge between paradigms, enabling Lean Six Sigma to be adapted through a person-centred lens to support healthful, values-based improvement. Reproduced with permission from “The Person-centred Lean Six Sigma Model” by Seán Paul Teeling.

The Person-centred Lean Six Sigma (PCLSS) model (Figure 2) comprises eight interrelated components drawn from Lean Six Sigma practice: Voice of the Customer, Respect for Person, Observational Studies (known as Gemba, taken from the Japanese), Staff Empowerment, Quality as an Influencer, Core Values, First Principles, and Standardisation. These components are grouped into three categories — synergy, divergence, and mutual influence, representing how each relates to the principles of person-centredness. The model does not assume alignment between the two paradigms, but instead provides a structure to examine points of harmony, challenge, and integration.

The PCLSS model offers a reflective and practical resource for Lean Six Sigma practitioners seeking to apply process improvement through a person-centred lens. While it retains key terminology from Lean Six Sigma, each component is interpreted through a relational perspective that prioritises values-based improvement and collaborative engagement. The model invites users to consider where Lean Six Sigma principles may align with, diverge from, or be adapted to person-centred ways of working. For example, the term “Voice of the Customer” is a Lean Six Sigma term that is intentionally retained within the model, which is explicitly designed for use by LSS practitioners who wish to apply LSS through a person-centred lens. However, the model emphasises that when operating within person-centred contexts, the Voice of the Customer must be understood as the authentic, participatory, and inclusive engagement of those receiving and delivering care. This includes collaborative activities such as listening, co-design, shared decision-making, and enabling ownership of change. It asks practitioners to ensure that their engagement is relational, not transactional, and that improvement efforts are shaped by the lived experiences of patients, families, and staff (11, 14).

The overlapping space in the centre of the model, Quality as Influencer, represents the intentional integration of selected relational and technical logics. Rather than collapsing synergy and divergence, it offers a conceptual meeting point that prompts critical reflection on how quality is understood, prioritised, and enacted across paradigms. The model acknowledges that “quality” is not a fixed concept but one that is contextually constructed, ranging from measurable outcomes to shared values and lived experience (3, 11, 14). This complexity challenges practitioners to engage with both the methodological rigour of Lean Six Sigma and the relational depth of person-centred approaches. In doing so, the model reinforces that while tensions may arise, both paradigms ultimately seek to contribute to quality improvement, a shared goal embedded at the heart of this overlap (3, 11, 14).

The model was developed through a realist-informed methodological approach, drawing on a multi-stage inquiry into how Lean Six Sigma contributes to person-centred care and cultures in healthcare settings (3, 11, 12, 14). Initial Programme Theories (IPTs) were generated through a realist review of the literature and collaborative engagement with an expert panel (3). This panel included senior clinical managers, front-line nurses and allied health professionals, person-centred practice researchers, improvement facilitators, and international academic advisors with specialist expertise in both person-centred methodologies and Lean Six Sigma (3). The IPTs were tested across study sites through realist evaluation, using context–mechanism–outcome (CMO) configurations to identify patterns of how Lean Six Sigma interacts with relational working and cultural conditions (3). The resulting Programme Theories (PTs) were then synthesised into a conceptual understanding that directly informed the structure of the model. Specifically, these PTs revealed recurring areas of alignment, tension, and co-adaptation between Lean Six Sigma and person-centred principles, which shaped the grouping of model components into synergy, divergence, and mutual influence (3, 11, 14).

Throughout the development of the PCLSS model, ongoing learning was integrated into a university-accredited postgraduate education programme in Person-centred Six Sigma for healthcare professionals across Ireland (12). The programme aimed to support practitioners in using the model to lead local improvement initiatives rooted in both technical rigour and relational values. The education curriculum included training in Lean Six Sigma tools and frameworks, systems thinking, structured facilitation, Voice of the Customer, Gemba observations, and reflective dialogue. In addition, participants were guided to examine their own improvement practice (i.e., their facilitation) through the lens of person-centredness and to co-design change initiatives aligned to service values.

The model has been applied across 12 healthcare sites in Ireland, spanning acute, community, and integrated settings, demonstrating its adaptability and potential for supporting cultural transformation in diverse service contexts (14). It is also currently in use in 12 countries, where both teams and individuals have adopted it to enhance person-centred Lean Six Sigma improvement efforts.

### 1.3 Healthful cultures

McCance and McCormack (19) developed the concept of healthful cultures through their work on person-centred practice and its cultural outcomes (20). Healthful cultures are defined as “contexts that are energy-giving for the benefit of health and wellbeing” — environments where both those delivering and those receiving care can flourish (19). These cultures are underpinned by respect for the person, mutual understanding, and shared decision-making. Importantly, they extend beyond individual behaviours to system-wide values and relationships (1, 19).

**Figure 3:**
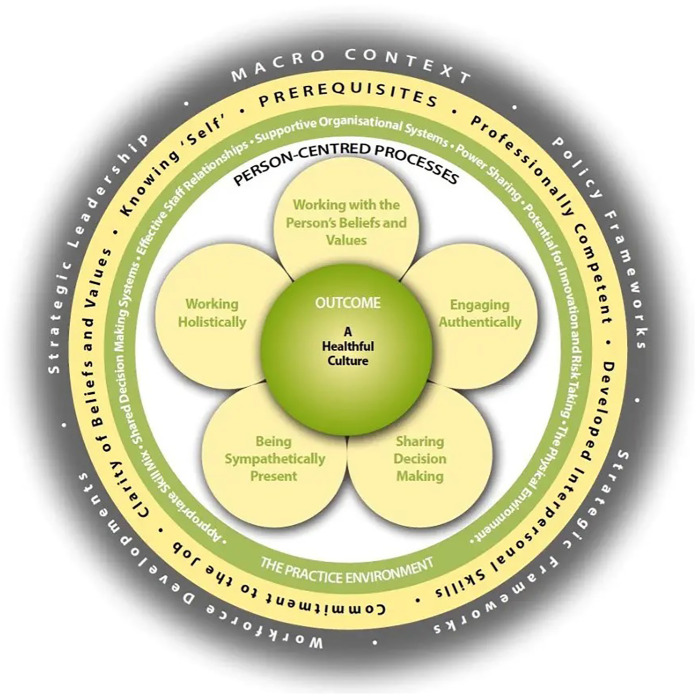
The person-centred practice framework source: This concentric model depicts the core domains that enable person-centred practice and the development of healthful cultures. At the centre are person-centred outcomes, supported by layers representing person-centred processes, the practice environment, staff attributes, and the macro context. Each layer is interdependent and connected, highlighting dynamic relationships between individuals, teams, and systems. The framework emphasises that person-centredness is shaped by contextual, relational, and structural factors, and that its enactment requires alignment across all levels of a healthcare system. Reproduced with permission from “Person-centred Practice Framework” by Brendan McCormack and Tanya McCance, licensed under CC-BY-NC-ND.

The Person-centred Practice Framework (Figure 3) (19) provides the basis for informing care-delivery models (21), curriculum frameworks (22), and research methodologies and practices (23). Over more than 20 years of research and practice development, McCormack and colleagues have identified key components of person-centred practice that contribute to the development of healthful cultures. These components include macro-contextual influences (strategic, political, and policy-related), staff attributes, the nature of the practice environment, person-centred processes, and person-centred outcomes. The framework highlights the complexity of healthcare systems and the dynamic interplay between individuals and structures.

A key characteristic of a healthful culture is that it does not prioritise the experience of people receiving care at the expense of staff wellbeing; rather, both are seen as interdependent. For cultures to be healthful, all persons must be energised by the context in which they work, and this energy must connect with the personhood of all involved (19).

While values such as compassion and kindness are important, McCance and McCormack (19) argue that they are not sufficient on their own to deliver person-centred practice. A shared language and system-wide understanding of person-centredness are essential to move beyond aspirational statements into practical, sustainable change. This aligns with global policy trends, including the World Health Organisation's (24) framework on people-centred healthcare, which emphasises integrated, strategic approaches to health system reform that centre on individuals, relationships, and local contexts.

### 1.4 Purpose of this paper

This paper seeks to explore the contribution of the Person-centred Lean Six Sigma (PCLSS) model to the development of healthful cultures in healthcare settings. While existing literature has articulated the importance of relational, person-centred approaches and the structured rigour of Lean Six Sigma, few studies have examined how these can be meaningfully integrated and applied to drive cultural change (3, 11). Notable exceptions include the work of Ward et al. (13), Teeling et al. (7) and Daly et al. (25), who have demonstrated the value of a whole-system, person-centred approach to improvement.

We propose that the PCLSS model provides a values-based, improvement-oriented framework that can support the cultivation of healthful cultures, as defined by McCance and McCormack (19, 20). To evidence this, we present four anonymised case studies of the model's implementation across diverse healthcare settings in Ireland. Through comparative analysis of the four studies, we examine how the model was used, what patterns emerged, and the extent to which its application aligned with the domains of the McCance and McCormack's work on Healthful Cultures.

This paper contributes to the ongoing discourse on how improvement methodologies can move beyond technical outcomes to embrace person-centred values, and how healthcare organisations can transition from isolated implementation to sustainable cultural transformation through reflective, participatory practice.

## 2 Methods

### 2.1 Study design

Sites were selected for inclusion based on three key criteria:

1. Staff who facilitated the improvement had completed training and education in the Person-centred Lean Six Sigma (PCLSS) model through a university-accredited education programme.
2. At the time of the study, the organisation was actively applying the PCLSS model to a defined improvement initiative/initiatives.
3. Senior management support was demonstrated through active leadership, resource allocation, and strategic alignment of improvement work with organisational goals, which aimed to deliver excellent, safe, quality, person-centred care.

A qualitative, instrumental multiple case study design was employed to explore the contribution of the PCLSS model to the development of healthful cultures within healthcare organisations. Instrumental case study designs enable an in-depth examination of a broader phenomenon through the investigation of multiple bounded cases (26). In this study, four healthcare sites were purposively selected based on their formal adoption and active implementation of the PCLSS model within organisational quality improvement initiatives. Each case was distinct in service type and context but shared the common feature of explicitly applying the PCLSS model to quality improvement activities.

• Study Site 1: Public Acute Teaching Hospital
• Study Site 2: Private Full-Service Acute Hospital
• Study Site 3: Integrated Community-Acute Ophthalmology Services
• Study Site 4: Public Rehabilitation Hospital

Each site applied the PCLSS model to one or more service improvement initiatives (Table 1).

**Table 1:** Summary of PCLSS model use by study site.

Study site	Improvement types	Time since organisational Lean Six Sigma deployment
Study Site 1: Public Acute Teaching Hospital	Dermatology outpatient access; Rehabilitation coordination	12 years
Study Site 2: Private Full-Service Acute Hospital	Surgical note documentation; Discharge pathway redesign; Outpatient access	8 years
Study Site 3: Integrated Community-Acute Ophthalmology Services	Referral redesign; Optometry-led care; Cataract surgery pathway	5 years
Study Site 4: Public Rehabilitation Hospital	Visiting policy redesign	3 years

Study Sites 1 to 3 implemented multiple projects across different service areas or clinical pathways, demonstrating the model's scalability and adaptability. For example, the Private Full-Service Acute Hospital and the Integrated Ophthalmology Services each addressed several distinct but complementary workstreams. In contrast, the Public Rehabilitation Hospital focused on a single, high-impact initiative related to visiting policy, reflecting its more recent adoption of the PCLSS model. Project focus, scope, and scale varied across settings, reflecting the flexibility of the PCLSS model to support both targeted and system-wide improvement. While a formal economic evaluation was not within the scope of this study, feasibility considerations were visible across sites. The model was applied in each site with implementation supported by locally available resources within existing quality or improvement departments. Costs primarily related to staff education and training in person-centred Lean Six Sigma, study leave to support this, and protected clinical time for project work. These were absorbed within organisational development budgets or supported through existing quality improvement initiatives, indicating that the model can be feasibly embedded in routine practice without requiring substantial external investment.

### 2.2 Data collection

#### 2.2.1 Documentary analysis

To support rigour and enable meaningful cross-site learning, documentary analysis and semi-structured interviews were carried out across all four sites. The research team obtained ethical approval to access qualitative and quantitative materials generated independently by local project teams during their implementation of the PCLSS model. These site-led projects were self-directed, with improvement aims designed and owned locally (see Table 1), and each site used the PCLSS framework to structure their design, implementation, and evaluation processes.

Each local team comprised interdisciplinary frontline healthcare professionals, including nurses, doctors, health and social care professionals, and administrative staff, all of whom had completed university-accredited education in Person-centred Lean Six Sigma (12). In the course of their work, these teams collected project-specific data using Lean Six Sigma tools and techniques, including Gemba observations, interviews, focus groups, and service user feedback (referred to as Voice of the Customer within Lean Six Sigma). These data were generated as part of each site's improvement activity and embedded in their reflective and evaluative practices.

The research team did not collect these data but instead undertook a structured documentary analysis of the existing material. This analysis aimed to examine how the PCLSS model had been applied in diverse, real-world settings, to identify contributions to healthful cultures, and to support cross-case insight and learning.

Documentary data reviewed included:

• Lean Six Sigma project documentation and outcome reports
• Site-generated surveys, interview transcripts, focus group summaries, and Gemba observation notes
• Staff reflective accounts and narrative feedback
• Presentations and internal evaluation reports prepared by local teams

#### 2.2.2 Semi-structured interviews

In addition to analysing site-generated documentation, the research team conducted semi-structured interviews with staff who had participated in the PCLSS initiatives (*n* = 16) across the four sites. A member of the research team, experienced in person-centred practice and quality improvement using Lean Six Sigma, but not involved in project delivery, conducted all interviews to support relational sensitivity, reflexivity, and openness.

An interview schedule was developed by the research team, structured around the eight core components of the Person-centred Lean Six Sigma (PCLSS) model: *Voice of the Customer, Respect for Person, Gemba (observational study), Staff Empowerment, Quality as an Influencer, Core Values, First Principles,* and *Standardisation*. These were mapped to the healthful culture domains identified by McCormack and McCance (19), including values-based leadership, inclusive practice, and staff well-being.

The schedule aimed to explore staff experiences of applying the model, while supporting critical reflection and values alignment. To ensure a person-centred orientation, the interview questions incorporated elements drawn from the Claims, Concerns and Issues (CCI) tool (27). This structure encouraged participants to identify what worked well, what challenges were encountered, and what uncertainties or questions remained.

For example, questions under *Voice of the Customer* included:

• “How were service users involved in your project?”
• “Did their involvement shape the design or implementation of changes?”
• “Were there any tensions or challenges in responding to feedback?”

Under *Staff Empowerment*:

• “Did you feel you were able to lead or initiate change?”
• “What supported or inhibited your ability to act?”
• “What would help strengthen staff empowerment in future initiatives?”

Similar exploratory questions were posed across the remaining domains to elicit both practical and cultural insights. Interviews concluded with open reflections, such as:

• “What impact, if any, did the project have on your team’s culture?”
• “How did the PCLSS model influence your experience of person-centred working?”

Each interview lasted 30–45min, was audio-recorded with participant consent, transcribed verbatim, anonymised, and analysed thematically. The integration of a person-centred approach and reflective orientation with research participants enabled the research team to explore both implementation outcomes and the underlying cultural and relational mechanisms that shaped them.

### 2.3 Data analysis

We drew on Braun and Clarke's (28) six-stage process of thematic analysis to guide the analysis of data within each case study, before drawing comparisons across cases. While the core stages were followed, the approach was adapted to suit the study's aims. Specifically, a hybrid inductive-deductive approach was adopted (29), allowing patterns to be generated directly from the data before being organised and interpreted in relation to McCance and McCormack's (19) work on Healthful Cultures. This adaptation enabled an open exploration of the data while also aligning emerging themes with an established body of work to enhance analytical depth and rigour.

The adapted process involved the following stages:

• Familiarisation: The research team first familiarised themselves with the collected data independently, then collectively, to support reflexivity and enhance analytical credibility (30). This included reviewing project documentation, reflective accounts, Voice of the Customer feedback, and observational data from Gemba, with repeated reading to develop a shared understanding of the material.
• Initial Coding: The data were coded by identifying meaningful units of information related to key aspects of person-centred practice, culture, and improvement outcomes. The coding process was inductive, generating codes directly from the data without pre-existing hypotheses (28). To ensure coding consistency, the research team collaboratively developed working definitions for key codes as they emerged. Initial coding was conducted independently by team members, followed by comparison and discussion to reach consensus on code application. Discrepancies were resolved through reflective team dialogue, supporting a transparent and reflexive approach to theme development. Data that did not align directly with Healthful Cultures was reviewed carefully; where appropriate, it was grouped under broader improvement outcomes related to service delivery. No significant data were excluded as redundant or unlabelled, ensuring a comprehensive account of the dataset.
• Generation of Themes: Through an iterative process, the research team grouped related codes into broader themes through comparison, discussion, and refinement (29).
• Review and Refinement of Themes: Once initial themes were established, they were reviewed for coherence and relevance through team discussions, incorporating feedback from all members. Themes were refined and redefined to ensure they accurately captured the complexity of the data.
• Mapping to Healthful Cultures: Themes were developed inductively from the case study data through open coding and iterative refinement. In the second stage of analysis, these emergent themes were deductively mapped onto the six domains of McCance and McCormack's (19) Healthful Cultures. This mapping process allowed the research team to explore the alignment between the themes generated from the PCLSS model's implementation and the principles of healthful cultures. The six domains used for mapping were: Leadership, Person-Centred Processes, Staff Empowerment, Collaborative Relationships, Supportive Practice Environments, and Shared Values and Vision. This two-stage approach ensured that the analysis remained grounded in the data while also linking the findings to an established theoretical model.

This process ensured that the analysis was grounded in the data and examined through a person-centredness theoretical framework, being rigorously examined to identify meaningful insights into the role of the PCLSS model in fostering healthful cultures in healthcare environments.

## 3 Results

This study explored how the Person-centred Lean Six Sigma (PCLSS) model contributed to developing healthful cultures across four healthcare settings. Findings are presented in three parts:

1. First, key improvements and impacts are outlined for each study site (Section 3.1).
2. Second, a cross-site comparative analysis identifies shared features of person-centredness and contextual adaptations (Section 3.2).
3. Third, these findings are mapped onto McCance and McCormack's (19) Healthful Cultures work (Section 3.3) to explore the contribution of the PCLSS model to developing healthful cultures.
4. Finally, we detail Quantitative Outcome Measures (Section 3.4).

### 3.1 Overview of findings from study sites

#### 3.1.1 Study site 1: public acute teaching hospital

This site focused on reducing waiting times for dermatology outpatient appointments and streamlining access to rehabilitation for older adults (5). Efficiency gains included reduced time to appointment, standardised triage, and coordinated discharge processes. Staff described a growing sense of control and reduced stress due to equitable workload distribution and system-level visibility. Patient confusion around care transitions was addressed through co-designed communication tools and simplified referral pathways.

#### 3.1.2 Study site 2: private full-service acute hospital

The PCLSS model was used at this site to improve service efficiency and care coordination. Examples included improving surgical note documentation (31), standardising discharge pathways (32), and enhancing outpatient access (33). Staff reported improved interdepartmental collaboration and a greater ability to contribute to meaningful change. The cultural shift was characterised by an increased sense of shared responsibility and respect for staff input.

#### 3.1.3 Study site 3: integrated community-acute ophthalmology services

This regional initiative bridged hospital and community services to enhance ophthalmology pathways. Use of the PCLSS facilitated the redesign of referral processes, increased optometry-led care, and improved access to surgery. The Voice of the Customer was central, with structured engagement of staff and patients. Reflective practice, collaborative learning, and shared ownership of change were integral to implementation, contributing to a strong sense of system-wide cohesion.

#### 3.1.4 Study site 4: public rehabilitation hospital

This hospital used the PCLSS model to redesign visiting policies, focusing on balancing safety, therapeutic goals, and person-centred values. Data-informed Gemba observations and surveys captured the voices of patients, staff, and visitors. Resulting changes included extended visiting hours, greater flexibility, and new guidelines that were co-designed and widely accepted. Staff reported reduced conflict, enhanced clarity, and improved morale. Patients and families experienced improved access, respect for preferences, and shared decision-making.

Across all sites, the PCLSS model enabled the co-creation of solutions tailored to local context, while reinforcing a broader cultural shift toward inclusivity, empowerment, and reflective practice.

### 3.2 Comparative analysis

#### 3.2.1 Commonalities across sites

Despite differences in setting, scale, and focus, several patterns emerged:

• Person-centred engagement: All sites emphasised the importance of Voice of the Customer and inclusive decision-making, resulting in interventions that reflected the real needs and values of persons delivering and receiving care.
• Empowerment and participation: Staff reported feeling more empowered and supported to initiate and lead change. The use of structured improvement initiative facilitation by qualified Lean Six Sigma practitioners was widely credited with fostering psychological safety and encouraging shared ownership.
• Sustainability and spread: Improvements were sustained and often led to second-generation projects. Sites developed mechanisms (e.g., dashboards, daily huddles, rotation of roles) to ensure continuity and adaptability.

#### 3.2.2 Differences in application

While the core model remained consistent, each site adapted its use of the PCLSS to its context:

• The Public Acute Teaching Hospital prioritised pathway coordination and clinical flow, with strong leadership from Lean Six Sigma-trained facilitators.
• The Private Full-Service Acute Hospital leveraged the model for cross-departmental redesign and data-driven innovation, particularly around documentation and discharge.
• The Integrated Ophthalmology Services initiative used the model for system-wide transformation, aligning hospital and community operations through co-designed regional governance.
• The Public Rehabilitation Hospital applied the model to enhance patient and family experiences, embedding relational practice into institutional policy and practice environments.

These variations affirm the model's adaptability, reinforcing its relevance across complex and evolving care contexts.

#### 3.2.3 Contextual enablers across sites

The PCLSS model demonstrated adaptability and success across the study sites, and several contextual enablers emerged that shaped the implementation process. These enablers were addressed through ongoing reflection and adaptation to the local context. Key enablers were:

• Leadership Engagement: Strong and consistent leadership commitment was identified as essential to the sustainability of improvement initiatives. Sites with robust leadership engagement demonstrated greater momentum, resource allocation, and staff empowerment, while variations in leadership commitment posed challenges to maintaining improvement efforts over time.
• Staff Engagement and Training: Staff participation varied, with some concerns raised regarding the balance between clinical responsibilities and improvement activities. Time constraints impacted engagement; however, flexibility in project scheduling and additional support helped address these challenges. In Sites 1, 2, and 3, dedicated improvement facilitators and/or colleagues who had completed the Lean Six Sigma education programme provided structured support to staff. In site 4, where dedicated improvement teams were not in place, support was provided by colleagues engaged in improvement initiatives, helping to maintain momentum and foster broader participation.
• Alignment with Organisational Culture: Integrating person-centred values with existing operational practices was a key enabler. The PCLSS model's emphasis on meaningful, non-volume-based metrics—such as care experience, responsiveness, co-designed processes, and relational quality (the empathy and strength of interactions between staff, patients, and families)—served as a useful conduit for aligning everyday practice with organisational culture and values.
• System Integration: Fragmented care pathways and communication challenges initially acted as barriers to improvement in some settings. These challenges were gradually addressed through increased collaboration across teams, iterative refinement of processes, and better integration of the PCLSS model into routine practice, ultimately enabling more coordinated and person-centred care.
• Implementation History and Cultural Readiness: Sites with longer-established Lean Six Sigma foundations demonstrated greater readiness for PCLSS implementation, showing more openness to participatory facilitation, reflective practice, and person-centred approaches. Familiarity with structured improvement methods, collaboration, and reflective learning contributed to the faster embedding of the PCLSS model. This reinforces broader findings that developing healthful, improvement-oriented cultures requires time to build trust, shared learning, and collective ownership of change (19, 34, 35).

Table 2 provides a high-level summary of how the shared features identified across the four study sites align with McCance and McCormack's (19) Healthful Cultures work.

**Table 2:** High-level summary of mapping to healthful cultures.

Healthful cultures domain	Study site 1: public acute teaching hospital	Study site 2: private full-service acute hospital	Study site 3: integrated community-acute ophthalmology services	Study site 4: public rehabilitation hospital
Leadership	Collective	Relational	Relational	Relational
Person-Centred Processes	Working with a person's beliefs and values Engagement Shared decision-making Providing holistic care	Working with a person's beliefs and values Engagement Shared decision-making Providing holistic care	Working with a person's beliefs and values Engagement Shared decision-making Providing holistic care	Working with a person's beliefs and values Engagement Shared decision-making Providing holistic care Sympathetic presence
Staff Empowerment	Co-designed care pathways	Staff engagement, training	System-wide collaboration, confidence	Empowerment in managing visits
Collaborative Relationships	Interdisciplinary collaboration, team trust	Cross-department collaboration	Interprofessional collaboration	Staff and family collaboration
Supportive Practice Environments	Psychological safety, reduced stress	Valued staff, involvement	Clear roles, staff engagement	Supportive policies, easy implementation
Shared Values and Vision	Patient flow, care coordination	Shared responsibility, teamwork	Access to care, integrated service delivery	Family involvement, therapeutic balance

### 3.3 Mapping shared features to healthful cultures

We now discuss these findings in greater detail, exploring how each site's unique context contributed to developing a healthful culture by applying the findings of the use of the PCLSS model to McCance and McCormack's (19) Healthful Cultures work.

#### 3.3.1 Leadership

The application of the PCLSS model strengthened relational leadership practices across all study sites, consistent with the Healthful Cultures (19). Leadership was found not to rely on hierarchical, top-down models, but instead demonstrated shared, enabling approaches that fostered trust, empowerment, and collective ownership of improvement work. Staff consistently described leadership as a relational process that supported meaningful engagement. A participant from site 1 noted, “The leadership during our project wasn't about one person telling us what to do … . it felt like we were all part of it. The … . model made leadership much more about connection and support”, highlighting the emphasis on inclusivity and empowerment. Similarly, a participant from Site 2 observed, “You could see the shift … . instead of directing us, leaders started asking us how they could support the changes we wanted to make”, reflecting a move toward enabling rather than directive leadership practices. This approach was reinforced at Site 3, where staff described, “PCLSS helped us see leadership as something shared. It wasn't top-down anymore; everyone had a voice, and that changed the culture completely”. One team member from Site 4 reflected on the relational nature of leadership, stating, “Leaders here now genuinely ask, “What do you think?” … less likely to hand down decisions”. These findings illustrate how relational leadership practices, as supported by the PCLSS model, helped create the enabling conditions necessary for the development of healthful, person-centred cultures across diverse healthcare settings.

#### 3.3.2 Person-centred processes

Across all study sites, the PCLSS model was found to support the embedding of person-centred processes, aligning with McCance and McCormack's Healthful Cultures work (19). Staff did not describe care as purely task- or process-driven; rather, there was a consistent emphasis on working collaboratively with service users to co-create meaningful care pathways. A staff member from Site 1 reflected this shift, stating, “We stopped designing services for clinical partners (General Practitioners) and started designing with them … the (PCLSS) work made that a real focus, not just a nice to have”, illustrating how service design became genuinely collaborative. Similarly, a participant from Site 2 observed, “(the model) helped us to look at processes through the patient's eyes, not just the service's needs … completely reframed how we work”, highlighting the refocusing of improvement priorities around individual experience. Staff at Site 3 described a move away from compliance-driven change, noting, “Before PCLSS, processes were about ticking boxes. Now, every change we make is about making things better for the individual at the centre”. This cultural shift was reinforced at Site 4, where one team member reflected, “The Voice of the Customer work opened our eyes … emphasis on patients and families shaping how we did things”. These findings demonstrate how the PCLSS model helped operationalise person-centred processes as core to system redesign rather than peripheral considerations.

#### 3.3.3 Staff empowerment

The PCLSS model contributed significantly to fostering staff empowerment across all study sites. Rather than improvement being driven exclusively by senior leaders, frontline staff were actively enabled to identify, test, and implement change initiatives, consistent with relational and enabling leadership approaches. A staff member from Site 1 noted, “I've never felt so trusted to make improvements … PCLSS didn’t just give us tools — it gave us permission to change things that didn’t make sense”, demonstrating the cultural shift toward distributed ownership of change. A participant from Site 2 similarly reflected, “Once we learned the method, it gave us real confidence … we weren’t just waiting for someone else to fix problems anymore”, highlighting the growth in individual agency. Staff at Site 3 described the dynamic nature of this empowerment, sharing, “Instead of waiting for approval … we felt empowered to test small changes ourselves. That energy spread through the team”. One team member from Site 4 emphasised how this empowerment became embedded in daily work, stating, “PCLSS changed the culture from “that's management's job” to “we all have a role in making things better” … That's still growing now”. Collectively, these accounts illustrate how the PCLSS model activated mechanisms of empowerment that were essential to sustaining person-centred improvement efforts.

#### 3.3.4 Collaborative relationships

Collaboration across professional groups and departments was consistently strengthened through applying the PCLSS model across all sites. Staff were not found to work in isolated silos but instead engaged in sustained, interdisciplinary collaboration aligned with the Healthful Cultures. A staff member from Site 1 explained, “We used to work in silos …  through PCLSS, we started genuinely collaborating across departments …  it felt like everyone was pulling together”, describing a tangible cultural shift. A participant from Site 2 reflected, “Working with other teams wasn’t an add-on anymore … it became part of how we solved problems, right from the start”, emphasising how cross-functional working was normalised. Staff at Site 3 noted the re-establishment of meaningful connections, commenting, “I didn’t realise how disconnected we were … until we started co-designing pathways. Suddenly, conversations opened up everywhere”. One team member from Site 4 similarly observed, “PCLSS taught us that no one group has all the answers … Collaboration became our new normal”. These reflections underscore how the PCLSS model helped dismantle professional silos and foster genuinely collaborative relationships in support of sustainable improvement.

#### 3.3.5 Supportive practice environments

The PCLSS model was found to contribute to the development of supportive practice environments characterised by psychological safety, shared learning, and open communication across the study sites. Rather than environments marked by fear of blame or rigid hierarchies, staff described work settings where reflection and improvement were encouraged. A staff member from Site 1 highlighted this change, stating, “There's more a sense of psychological safety now … it's okay to say, “This isn’t working” without fear. That started with the PCLSS approach”. A participant from Site 2 echoed this, observing, “Even small changes to how we meet and reflect … made work feel safer and more open … people felt heard”. Staff at Site 3 reinforced the role of psychological safety in cultural change, sharing, “Before, raising problems felt risky …  Now it feels expected — and that's made a huge difference to morale”. One team member from Site 4 illustrated how positive reinforcement supported change, noting, “Small improvements like celebrating quick wins …  made work feel more energising. It wasn’t all about targets anymore”. These findings highlight the critical role of supportive environments in enabling healthful cultures, with the PCLSS model serving as a catalyst for their development.

#### 3.3.6 Shared values and vision

A clear sense of shared values and collective purpose was consistently evident across all study sites following the application of the PCLSS model. Rather than being driven by disparate departmental agendas or compliance-focused targets, teams articulated a unified commitment to person-centred improvement. A staff member from Site 1 described this shift, stating, “We’re no longer chasing random KPIs … the work helped us build improvement goals that reflect what matters to patients and staff”. A participant from Site 2 similarly reflected, “The model helped us agree on what “good care” actually looks like … now everyone's aiming for the same things, not just following checklists”. At Site 3, staff described how these shared values permeated onboarding practices, sharing, “Even new staff pick up on the culture straight away … that wouldn’t have happened without the clarity we gained through the (PCLSS) model”. One team member from Site 4 emphasised the significance of this cultural embedding, noting, “The biggest change is we now have a common purpose that guides what we do every day …  not just a poster on the wall”. Collectively, these reflections illustrate how the PCLSS model supported the articulation and enactment of shared values and vision across diverse healthcare settings.

### 3.4 Quantitative outcome measures

The preceding thematic analysis, mapped to McCormack and McCance's Healthful Cultures work, demonstrated how the PCLSS model supported the development of healthful cultures across a range of healthcare settings. Key themes included shared values and vision, relational leadership, and supportive environments that empowered staff and prioritised person-centred care. To complement these qualitative insights, we now present verified quantitative outcome data from each study site. These data illustrate how the application of the PCLSS model was associated with measurable improvements in access to care, service efficiency, and stakeholder experience.

At Study Site 1, a large public teaching hospital, improvements to referral, triage, and waiting list management in a dermatology service led to a reduction in total outpatient waiting list numbers from 3,736 in September 2020 to 2,228 by June 2021 (a 40% reduction). The number of patients waiting over 12 months for an appointment decreased by 60% (from 1,615 to 634). Mean wait times fell across all triage categories, including a 61% reduction in the “Urgent” category (from 118 to 45 days), 70% in the “Soon” category (517 to 155 days), and 32% in the “Routine” category (358 to 241 days). The Mann–Whitney *U*-test confirmed a statistically significant reduction in waiting times post-intervention (*p* < 0.001) with a median decrease of 169.95 days (5).

At Study Site 2, a private full-service acute hospital, improvements that were implemented included system wide work to support the safe and person-centred resumption of services following COVID-19 restrictions. Comparing July–December 2020 to the same period in 2019, inpatient admissions increased by 6%, inpatient surgeries by 21%, and outpatient surgeries by 4%. These gains occurred despite reduced activity earlier in the year due to lockdown. Patient satisfaction rose from 93% to 95%, and notably, there were zero reported cases of in-hospital COVID-19 transmission from March to December 2020 (25).

At Study Site 3, an integrated care initiative introduced a model for immediate sequential bilateral cataract surgery (ISBCS). This led to a 66% increase in surgeries completed on half-day lists (from 6 to 10 per list), while mean turnover time between surgeries was reduced from 13.8 to 8.7min. Patient visits were reduced from five to three per episode of care. Each bilateral surgery delivered cost savings of €450 (direct), €400 (indirect), and reduced patient travel by approximately 167 km—saving 1 tonne of CO₂ for every 30 surgeries performed.

At Study Site 4, a public rehabilitation hospital, a person-centred visiting policy was co-designed with staff, patients, and families. Surveys indicated strong support for increased flexibility, privacy, and inclusivity. The new policy incorporated extended visiting hours, access for children, and greater use of shared spaces. Staff feedback indicated greater ease in implementing the revised policy and increased feelings of being heard and respected. Over 90% of patients and visitors and 75% of staff supported proposals to open the hospital canteen in the afternoon to further improve the visiting experience.

Together, these outcomes demonstrate the potential of the PCLSS model to generate measurable service improvements while upholding person-centred values. Quantitative gains were achieved in areas such as access, efficiency, safety, satisfaction, environmental impact, and staff empowerment. These results underscore the practical value of aligning Lean Six Sigma methodologies with relational principles to drive meaningful, sustainable change in healthcare. The implications of these findings are explored further in the following Discussion.

## 4 Discussion

### 4.1 Embedding healthful cultures through the PCLSS model

The findings of this study demonstrate that the Person-centred Lean Six Sigma (PCLSS) model is not only adaptable across diverse healthcare contexts but also strongly aligned with the development of healthful cultures. By synthesising the technical structure of Lean Six Sigma with the relational principles of person-centredness, the model offers a credible pathway to sustainable culture change within healthcare systems (3, 14).

### 4.2 Leadership as an enabler of sustainable improvement

Leadership practices emerged as critical to the success and sustainability of the PCLSS model. Leaders who modelled person-centred values, facilitated staff empowerment, and prioritised authentic communication (34) were identified as essential enablers of sustained improvement. Conversely, where leadership engagement was absent or transactional, teams faced challenges in embedding change. These findings reinforce the importance of leadership approaches that enable relational cultures and continuous improvement. The PCLSS model is consistent with leadership principles used in Lean Six Sigma improvement initiatives, which emphasise leadership commitment, daily management systems, and continuous staff engagement to drive performance improvement. Shortell and colleagues (35) found that hospitals with established Lean implementations—characterised by strong leadership involvement and structured daily management practices—reported more positive performance outcomes. The PCLSS model cultivates the leadership behaviours essential for sustaining Lean initiatives and embedding a culture of continuous, person-centred improvement by fostering participatory facilitation and inclusive dialogue. Relational leadership can act as a catalyst for organisational readiness, alignment with values, and investment in staff training, which were identified as key contextual factors influencing the extent of success and sustainability of the model.

### 4.3 Addressing philosophical tensions between Lean Six Sigma and person-centredness

While early literature on Lean Six Sigma in healthcare highlighted philosophical tensions, such as privileging efficiency over individual experience (36), there has been limited empirical engagement with how such tensions might be meaningfully addressed. Our earlier realist review found that only a small number of studies referenced both paradigms, and fewer still examined their intersection in depth (3, 4, 11, 37). As a result, a significant gap exists in understanding how person-centred principles and Lean Six Sigma practices can be integrated to support sustainable improvement. Our ongoing programme of research (3–7, 9, 11, 13, 31–33, 38) seeks to address this gap through empirical, realist, and applied work.

In doing so, we have also drawn on wider critiques of Lean implementation in healthcare. For example, Kaplan et al. (39) discuss failures of alignment between technical models and complex healthcare settings. Dixon-Woods et al. (40) and Flynn et al. (41) similarly warn against adopting improvement methodologies without attending to relational and cultural dynamics. These perspectives have shaped the development of the Person-centred Lean Six Sigma (PCLSS) model, which seeks not to eliminate tensions between paradigms, but to make them visible and usable through intentional reflection and design.

As outlined earlier, the model presents eight Lean Six Sigma components structured into domains of synergy, divergence, and mutual influence, offering a framework to explore both alignment and conflict. Crucially, the central overlapping space, Quality as Influencer, was conceived not as a point of philosophical fusion, but as a critical site of negotiation. It acknowledges that “quality” is not universally defined: it may refer to measurable outcomes, values-based experiences, or culture change (3, 11, 14). By positioning quality as a dynamic and contextually shaped concept, the model supports reflective adaptation of Lean Six Sigma through a person-centred lens.

What is reiterated here is the model's intended function as both a practical tool and a philosophical provocation. Rather than offering a prescriptive solution, the PCLSS model encourages practitioners to navigate methodological rigour and relational responsiveness side by side. It invites users to examine how quality is interpreted in their own settings, and how Lean Six Sigma tools, such as Voice of the Customer or Gemba, might be enacted in more participatory and inclusive ways. In this way, the model becomes a structured means of exposing and working with tensions, rather than denying them.

This study, which applied the model across diverse clinical settings, four of which are presented in this paper from a wider pool of twelve evaluation sites, enabled further testing of this reconciliation. Our findings show that, when guided by shared values and relational leadership, the integration of technical and relational work not only mitigates philosophical tensions, but also creates the conditions for healthful cultures to flourish.

### 4.4 Demonstrating the application of the PCLSS model in diverse settings

In this paper, we illustrated how the PCLSS model was applied in four diverse healthcare settings as part of a broader twelve-site programme of work. This study builds on previous concerns by testing a deliberately person-centred reframing of Lean Six Sigma that integrates relational principles into technical structures. Through this application, we explored how aligning person-centred and improvement paradigms could create the conditions for healthful cultures to flourish.

### 4.5 Participatory practice, relational leadership, and cultural change

A core strength of the PCLSS model lies in its capacity to enable meaningful staff engagement at all levels. Rather than imposing externally driven, top-down change, the model supports co-creating local solutions through participatory facilitation, reflective practice, and inclusive dialogue (5, 25, 32). The university-accredited education programme, which embedded the PCLSS model into staff development, leadership practice, and systems thinking, was previously identified as a significant enabler of participatory approaches in earlier research (12, 38). Comparative analysis across all four sites in this study further reinforces these findings, demonstrating the central role of structured education in embedding person-centred improvement practices and fostering cultural change. This approach nurtures psychological safety, builds interprofessional trust, and encourages systems thinking, all of which are fundamental to sustaining improvement cultures (13, 34). The study highlights that technical tools alone are insufficient to achieve meaningful and lasting change (3, 12), and this tools-based approach may not actually achieve culture change (42). It appears that the integration of relational and technical work in the approach facilitators of change take, embedded within the PCLSS model, activates the mechanisms needed to cultivate and sustain healthful workplace cultures in healthcare systems. Mapping the findings to McCance and McCormack's (19) Healthful Cultures work confirms that improvements enacted through the PCLSS model extend beyond technical process optimisation. Engagement with the PCLSS helps transform leadership practices, strengthen relational connections, and positively impact the lived experiences of staff, patients, and families. Healthful cultures are characterised by shared values, collaborative working, empowered decision-making, and collective leadership (19), all consistently evident across the study sites.

### 4.6 Alignment with established culture frameworks

Importantly, these findings also align with Manley and colleagues' (43) conceptualisation of effective workplace cultures. The PCLSS approach reflects the enabling factors identified by Manley et al., such as collective leadership, skilled facilitation, shared values, and supportive learning environments. By fostering workplace cultures where staff feel valued, psychologically safe, and engaged in collaborative improvement, the model contributes directly to both system performance and human flourishing. Notably, each site reported a ripple effect, where initial improvement projects catalysed further innovation and ongoing development of a person-centred improvement culture. This reflects the dynamic and evolving nature of healthful cultures, which are sustained through experiences of success, relational connectedness, and the visibility of shared achievements (19).

### 4.7 Limitations and generalisability

While the findings offer transferable insights for improvement practice, this study is contextually bounded in scope. The PCLSS model was deliberately developed to support healthcare practitioners and teams to apply Lean Six Sigma in a person-centred manner. While it is applicable across a wide range of healthcare settings, including public and private, as well as acute and community services, it is specifically intended for use by those employing Lean Six Sigma. However, as noted, Lean Six Sigma has become one of the most widely adopted methodologies in international healthcare improvement practice (3, 10), and the PCLSS model has now been translated into German and Spanish, with use reported across 12 countries. This reflects its growing accessibility and practical relevance for diverse health systems seeking to embed person-centredness within technical improvement work. Rather than offering a rigid toolkit, the model provides a flexible framework that can be locally adapted while remaining grounded in its core principles. This paper extends the evidence base for the model, demonstrating its contribution to cultivating healthful, person-centred cultures through the intentional integration of technical and relational approaches.

While the model demonstrated feasibility across four diverse settings, including public acute, private acute, community hospital, and rehabilitation care, this study did not include a formal analysis of implementation costs or long-term scalability, with all study sites at different stages of implementation (Table 1). Costs were primarily associated with staff study leave, protected staff time for project work, and access to education in Person-centred Lean Six Sigma. However, organisations reported the outcomes detailed in Section 3.4 as representing a visible return on investment (ROI), particularly through improvements in service processes, patient experience, and team culture. These perceived benefits, alongside high levels of staff engagement and leadership in local improvement efforts, contributed to the model's acceptability and uptake. Future work may benefit from dedicated economic evaluation and exploration of model adaptation in lower-resource systems.

We acknowledge that the literature cited in this study includes a concentration of work by the lead authors and their close collaborators. This reflects the relatively limited body of research to date that directly explores the intersection of Lean Six Sigma and person-centred care. Rather than indicating insularity, this underscores the originality of the work and the emerging nature of this field. The development of the PCLSS model has been shaped through a cumulative programme of realist review, realist evaluation, and applied research (3, 11, 14), which explicitly engages with wider theoretical and critical perspectives. These foundational studies have laid the groundwork for further testing and development by a broader range of research teams in varied contexts.

## 5 Conclusion

This study demonstrates that the PCLSS model offers a distinctive and sustainable approach to healthcare improvement by integrating technical excellence with relational, person-centred values. Across four diverse healthcare settings, the PCLSS model supported the development of healthful cultures in ways that reflect the enabling factors including transformational leadership, skilled facilitation, shared values, and supportive learning environments (19, 42). Specifically, staff were empowered through education, facilitation, and active participation, aligning with the creation of supportive learning environments; collaborative relationships were built through interdisciplinary engagement and co-design, underpinned by shared values; shared leadership emerged through distributed responsibility and inclusive decision-making, supported by transformational leadership; and system-wide trust was fostered through consistent facilitation, reflective practice, and transparent communication. These interconnected elements demonstrate how the PCLSS model operationalises the relational and technical conditions needed to embed a person-centred, improvement-focused culture.

A critical finding was the importance of cultural readiness in shaping the successful adoption and impact of the model. Sites with a longer history of structured improvement work displayed greater openness to participatory approaches and a stronger capacity to embed person-centred processes. This reinforces that building healthful, sustainable cultures is a dynamic, evolving process that requires time, leadership commitment, and shared ownership of change (44).

By fostering participatory facilitation, reflective practice, and inclusive dialogue, the PCLSS model activates the mechanisms needed to sustain improvement over time. It enables organisations to move beyond a narrow focus on process optimisation, creating environments where both staff and patients can flourish. Ultimately, the PCLSS model makes a distinctive contribution to the creation of healthful cultures—cultures where relational, technical, and organisational practices align to support human flourishing as a central goal of healthcare improvement.

## Data Availability

The datasets presented in this article are not readily available without access through the corresponding author commensurate with Ethical approval. Requests to access the datasets should be directed to sean.p.teeling@ucd.ie.
